# PROTOCOL: Interview and interrogation methods and their effects on true and false confessions: An update and extension

**DOI:** 10.1002/cl2.1314

**Published:** 2023-03-02

**Authors:** Mary Catlin, David B. Wilson, Allison D. Redlich, Talley Bettens, Christian A. Meissner, Sujeeta Bhatt, Susan Brandon

**Affiliations:** ^1^ Criminology, Law and Society George Mason University Fairfax Virginia USA; ^2^ Department of Psychology Iowa State University Ames Iowa USA; ^3^ Institute for Defense Analyses Alexandria Virginia USA; ^4^ SyncScience New York New York USA

## Abstract

This is the protocol for a Campbell systematic review. The objective is to assess the effects of interrogation approach on confession outcomes for criminal (mock) suspects.

## 1 BACKGROUND

### 1.1 Description of the problem

In 1999, Victoria Bell Banks convinced law enforcement to release her from custody after claiming to be pregnant. Later, when the Choctaw County Sheriff came looking for a baby, Victoria claimed she had a miscarriage. The sheriff was suspicious and eventually began questioning Victoria's estranged husband about the infant. After several days of intense interrogation, Medell Banks falsely confessed to the child being born alive and buried near his property. It was not until medical doctors examined Victoria and determined that she was physically incapable of being pregnant that Medell was exonerated (NRE, 2021). It feels surprising that someone would confess to a crime they did not commit (Leo, 2009), let alone a crime that never took place. Researchers, however, have long recognized the problematic nature of false confessions (e.g., Münsterberg, 1908).

False confessions pose several problems for the criminal legal system (e.g., Kassin, 2014; Scherr et al., 2020). First, it is very difficult to distinguish between true and false confessions. For example, one set of researchers asked a group of prisoners to tape themselves providing a true confession to a crime they had committed and been convicted of and a second false confession to a crime that the researchers had fabricated. These tapes were then presented to both college students and trained police officers who were asked to identify whether the confession they were viewing was true or false. Unfortunately, while the police officers claimed to be more confident in their judgments about the veracity of the confessions, they were no more accurate than the college students and both groups overall performed poorly (Kassin et al., 2005). Confessions—whether true or false—are so powerful that they tend to persuade juries and even judges more than any other piece of evidence. Mock jury studies have found that confessions are seen as more inculpatory than any other form of evidence (e.g., Kassin & Neumann, 1997) and that even when individuals are informed that a confession was coerced and should legally be disregarded, the confession still influences their guilt decisions (Kassin & Sukel, 1997).

Even if the confession were deemed inadmissible and completely disregarded by the legal actors asked to make a final determination of guilt, the confession could still impact perceptions of the case through other evidence. Specifically, research examining forensic confirmation biases (see Kassin et al., 2013) has found that knowing a confession has been obtained can impact forensic experts charged with determining whether fingerprints match (Kukucka & Kassin, 2014) or even the conclusions of DNA tests (Dror & Hampikian, 2011). The undue influence of confessions on other evidence creates two problems: (1) legal actors could be influenced by a (false) confession even if they do not realize it and (2) law enforcement conducting the investigation are unintentionally increasing their own confirmation biases. Specifically, once a suspect confessesions and the other available evidence begins lining up with that same suspect, the chances become reduced that law enforcement will use limited resources to continue pursuing other potential leads. The snowball effect that a false confession can create is part of the framework discussed in the Cumulative Disadvantage Framework (Scherr et al., 2020). Once a false confession occurs, several cognitive processes activate that can culminate in a wrongful conviction. First, when an innocent suspect confessesions, law enforcement is less motivated to track down other leads and may turn their attention almost exclusively to the suspect who confessed. That confession then taints other forensic evidence making the innocent suspect appear even more guilty. If the innocent suspect does not plead guilty first, at trial it is unlikely that jurors will be able to forget the confession evidence once it is introduced, even if instructed to ignore. Regardless, in both plea and trial settings, the confession will serve as the single most powerful piece of evidence. It comes as no surprise then that individuals end up wrongfully convicted because of a false confession (Scherr et al., 2020).

In fact, of the over 3000 exonerations, estimates from the NRE suggest that 12% of all known wrongful convictions involved a false confession (NRE, 2020)—like Medell Banks. Beyond the false confession, wrongful convictions also mean that the true perpetrator has not been identified. In fact, in one estimate, researchers suggest that an additional 41,000 crimes a year are committed by the true perpetrators in cases where someone has been wrongfully convicted (Norris et al., 2020). Thus, wrongful convictions can produce two harms: an innocent individual is punished for a crime they did not commit, and a guilty offender remains unpunished and often at large in the community (see Norris et al., 2020). A key driver of wrongful convictions is false confessions, which almost exclusively occur during interrogations, and have been shown to be influenced by the interrogation method used. Taking stock of the evidence on the relative performance of the two dominant interrogation methods (i.e., accusatory and information gathering) can inform police department policies and training regarding interrogation methods and the broader public debate around police reforms.

### 1.2 Description of the intervention

False confessions are the product of an interrogation process, and the method by which an interrogation is conducted likely affects both the rate of truthful confessions and false confessions. An optimal interrogation method will maximize the former and minimize the latter. While there are endless variations in how an interrogation may be conducted, the two general styles are an accusatory approach and an information‐gathering approach (Kelly et al., 2013). The proposed meta‐analysis will focus on accusatorial and information‐gathering approaches to interrogations, briefly described in the following paragraphs, as they are the most popular approaches.

First, the Reid technique is often referred to as *the* exemplar of an accusatorial approach. This most common interrogation manual taught to law enforcement in the United States starts with an assumption of guilt and relies on law enforcement's ability to detect deception—typically through nonverbal behavior like avoiding eye contact. The goal of the interrogation is to obtain a confession from a suspect (Inbau et al., 2013), focusing on minimization (e.g., suggestions of leniency, justifications, and rationalizations) and maximization (e.g., refusing to accept denials, exaggerating the severity of the situation, exaggerating and/or fabricating evidence) tactics (Kassin and McNall, 1991). For a more complete discussion of accusatorial tactics, see Kelly et al., 2013.

Second, just as Reid is the examplar of an accusatorial approach, PEACE has become the primary example of an information‐gathering approach. Facing concerns over false confessions and their possible connection to an accusatory interrogation method, Great Britain passed the Police and Criminal Evidence Act (PACE) of 1984. The goal of PACE was to move away from accusatory interrogation techniques that closely resembled those used in the United States. In contrast, the PACE system's goal for the interview is to gather information through techniques such as rapport building, open‐ended questioning, and encouraging suspects to speak freely (Bull & Milne, 2004). Many other countries, such as Australia, New Zealand, and Norway have adopted this information‐gathering approach. Stemming from PACE, a new standard of investigative interviewing was formed and has since become the standard alternative to accusatorial interrogation approaches: PEACE. PEACE is the structure of all interviews—regardless of the interviewee's status in the investigation—including the Planning and Preparation of the interview, Engaging the interviewee and Explaining the ground rules of the interview process, obtaining an Account, Closure of the interview, and Evaluation of the interview process (see Milne et al., 2007 for an overview). See Gabbert et al. (2021) for a systematic review of rapport techniques. Further, there is research conducted by the High Value Detainee Group (HIG) that has led to an additional model of rapport‐based information gathering (see Meissner et al., in press).

This alternative approach to accusatorial interrogations raises the question of whether these information‐gathering techniques would reduce false confessions without reducing true confessions.

### 1.3 How the intervention might work

The interrogation is the setting of most confessions, with researchers suggesting that the interrogation approach could be directly responsible for false confessions (Ofshe and Leo, 1997). Specifically, two factors could influence whether an interrogation will result in a false confession from an innocent person: (1) if interrogators enter an interrogation with an a priori assumption of the suspect's guilt and (2) how rapport is used during the interrogation. Assumptions of guilt can be problematic because interrogators who believe the (innocent) suspect is guilty are more likely to induce a false confession because they are more likely to use coercive interrogation tactics (Narchet et al., 2011). Theoretically, then, interrogation approaches that encourage assumptions of guilt will be more likely to induce false confessions, while interrogation approaches that avoid assumptions of guilt should minimize false confessions. According to the Cumulative Disadvantage Framework (CDF; Scherr et al., 2020), false confessions are a natural consequence of accusatorial interrogations (Mortimer & Shepherd, 1999). More specifically, the CDF framework argues that the assumption of guilt triggers a series of actions that can lead to a false confession because of confirmation biases. In turn, a false confession can influence the interpretation of forensic evidence by investigators, suggesting even more “inculpatory” evidence than existed before the false confession (e.g., Kassin et al., 2013). From there, the chances of innocents accepting a plea deal or being convicted at trial increase dramatically (e.g., Appleby & Kassin, 2016; Leo, 2009). Even if exonerated, the false confession holds severe consequences for exonerees attempting to reenter society (e.g., Kukucka & Evelo, 2019).

In contrast, the information‐gathering approach is argued to reduce false confessions by avoiding presumptions of guilt. Information‐gathering approaches, like PEACE, do not start with an assumption of guilt. Theoretically, by changing the goal of an interview from obtaining confessions to information, these techniques avoid the harmful actions associated with the confirmation bias in interrogation settings (e.g., CDF; Scherr et al., 2020).

Assumptions—or lack thereof—of guilt could also influence the use of rapport during interrogations. Rapport, broadly defined, is the connection established between the interviewee and the interviewer. Rapport tactics are typically intentional behaviors employed by interviewers to encourage information disclosure from interviewees. These tactics can be verbal, para‐verbal, or non‐verbal, though the most common tactic in the literature is active listening. Importantly, information‐gathering approaches recognize that rapport is not a static characteristic and can change over the course of an interaction (Alison & Alison, 2017). In contrast, the Reid technique only discusses rapport at the start of an interview phase (Inbau et al., 2013). This approach is problematic because rapport can, and likely will, wane as more accusatorial approaches are introduced to the relationship. Furthermore, by itself, rapport can be used as a form of minimization by falsely leading interviewees to believe the interviewer is working in their best interest (David et al., 2017). By addressing rapport throughout the interview, information‐gathering approaches should enhance interviewee cooperation, which in turn should produce more reliable information (Vanderhallen & Vervaeke, 2014). In fact, research has shown that interviewers who are able to establish and maintain rapport are more likely to obtain favorable outcomes (Walsh & Bull, 2012). When interviewers use rapport tactics, especially those aimed at aligning the interviewee with the interviewer, interviewees are more likely to perceive rapport. The perceived rapport increases cooperation, which in turn increases the amount of information disclosed by the interviewee (Brimbal et al., 2019, 2021; Dianiska et al., 2021). Thus, because information‐gathering approaches both avoid assumptions of guilt and actively build rapport throughout interrogations, this approach should result in fewer false confessions than accusatorial approaches that do neither.

### 1.4 Why it is important to do this review

Meissner and colleagues (Meissner et al., 2012) conducted a Campbell systematic review and meta‐analysis investigating rates of true and false confessions across information‐gathering, accusatorial, and direct questioning (i.e., control) interrogation techniques across both field and experimental (laboratory) studies. The synthesis of the field‐based studies found that both information‐gathering and accusatorial interrogation techniques were more likely to elicit a confession when compared to direct questioning. These findings are limited, however, because field studies cannot establish ground truth (actual guilt or innocence). As such, estimated rates of false versus true confessions are not possible, only the overall effectiveness of the method at getting a confession (regardless of its reliability). In contrast, the findings from Meissner et al.'s (2012) systematic review of the experimental studies suggested that while both information‐gathering and accusatorial approaches increased the number of confessions as compared to control conditions (i.e., direct questioning), accusatorial approaches also increased the number of false confessions when contrasted directly with information‐gathering approaches. The authors cautioned strong claims regarding the comparison of the two approaches because the analysis was based on small sample sizes and they urged future researchers to more explicitly compare information‐gathering and accusatorial techniques (Meissner et al., 2012).

This review is a partial update of Meissner and colleagues' (Meissner et al., 2012) review, focusing solely on the experimental studies. It is important to focus on the experimental studies as only experimental studies can speak to the diagnosticity (i.e., the ability to maximize true confessions while minimizing false confessions) of interrogation approaches. By their nature, experimental studies cannot exactly mirror interrogations that happen in the real world. The tactics and scripts used in experimental studies, however, are based on real‐world interrogation manuals (e.g., Inbau et al., 2013), induce meaningful physiological and psychological changes in the participants accused (e.g., Guyll et al., 2019; Normile & Scherr, 2018), and focus on the same underlying psychological processes that exist in real interrogations. Thus, it is reasonable to use experimental studies as analogues for real‐world practice to benefit from the implications the associated diagnosticity information can have on policy and practice. The results of the proposed partial update will not only be able to speak to whether the legal system should continue relying on accusatorial techniques but will also be able to speak to the growth of accusatorial tactics across contexts. Specifically, the Reid corporation has expanded their practices beyond the interrogation room to other settings such as high schools, Child Protective Service offices, and so on. The results of this study will provide policymakers and practitioners with evidence‐based research to indicate whether accusatorial approaches produce an increase in false confessions.

To our knowledge, there have been three other meta‐analyses looking at interview and interrogation research in the time since 2012. The more recent effort used meta‐analytic approaches to examine the effect of rapport‐building and support tactics on children's disclosure in forensic interviews (Lavoie et al., 2021). The focus on child witnesses, however, limits the applicability of these results to the proposed update (which focuses solely on suspects). The second meta‐analysis is more pertinent to our efforts as it examined the prevalence of false confessions across experimental paradigms. Results indicated that typing task studies (i.e., the alt‐key paradigm) were the most likely to result in false confessions regardless of typing speed. Furthermore, compared to all other tactics, false evidence ploys (i.e., lying or bluffing to suspects about evidence) were more likely to result in false confessions (Stewart et al., 2016). The last meta‐analysis examined the social, cognitive, and affective factors associated with true and false confessions obtained using the cheating paradigm. Results demonstrated that false confessions were associated with perceptions of the consequences of confessing and perceptions of the interrogation context (Houston et al., 2014). None of these meta‐analyses, however, looked at the impact of interrogation approaches on suspect false confessions. Therefore, updating Meissner and colleagues' (Meissner et al., 2012) review is important, paying particular attention to experimental paradigm and accusatorial tactics. We have conducted a pilot search based on the proposed methodology, and can confirm there exist sufficient numbers of new studies (~8) to warrant an updated review. These additional lab‐based studies conducted since 2010 and advances in the methods of meta‐analysis over the past decade, such as the development of network meta‐analysis and associated software implementations, will extend prior synthesis work in this area.

## 2 OBJECTIVES

The current study is a partial update and extension of Meissner and colleagues' (2012) prior Campbell systematic review titled *Interview and Interrogation Methods and their Effects on True and False Confessions*, focusing solely on experimental or laboratory‐based studies. Our objective is to assess the effects of interrogation approach on confession outcomes for criminal (mock) suspects.

To address our objective, a series of meta‐analyses will be conducted, contrasting accusatorial, information‐gathering, and direct questioning interrogation approaches on their ability to elicit true and false confessions. Like its predecessor, the proposed meta‐analysis will focus on (mock) suspects as the population of interest, interview style as the intervention, and the diagnosticity (i.e., ability to increase true confessions while minimizing the number of false confessions) of the interview styles as the indicator of effectiveness.

Furthermore, to expand on past work, the current effort also proposes a meta‐analysis contrasting two accusatorial tactics: minimization and maximization (including false evidence ploys). To accomplish this goal, a network meta‐analysis will be conducted to compare not only macro‐level interrogation styles but how different approaches within the accusatorial approach (i.e., minimization and maximization) compare to each other and to other schools of interrogation techniques.

## 3 METHODS

### 3.1 Criteria for considering studies for this review

All of the criteria for study inclusion are based on the original review by Meissner and colleagues (Meissner et al., 2012). However, we have noted where we plan to make departures from those original criteria.

#### 3.1.1 Types of studies

For this update, we will include only experimental studies, regardless of publication status, that randomly assign mock subjects (i.e., not real criminal justice suspects in field studies) to two (or more) interrogation (interview) conditions. The experimental manipulation must include the random assignment of an accusatorial or information‐gathering interrogation technique. The two techniques can be compared with each other, compared to a control interrogation technique (e.g., direct questioning), or for studies with only accusatorial techniques, include some contrast of minimization, maximization, and control tactics. Participants (mock suspects—see below) can be entirely aware of the nature of the study (e.g., some studies challenge participants to “get away” with an act of wrongdoing) or can be deceived to various degrees (e.g., some studies lead participants to believe they are facing academic consequences for the supposed wrongdoing). Any experimental paradigm is eligible (e.g., both cheating and alt‐key paradigms will be included) and studies can include more than one manipulated factor. However, manipulated factors not pertinent to our review will only be reported with the description of study characteristics, not analyzed.

The prior review also included field‐based observational studies. These will be excluded from this review as our objective focuses on the reliability of confessions, which is not possible to assess with field studies (where ground truth remains unknown).

#### 3.1.2 Types of participants

Participants will be mock suspects who were accused of some wrongdoing. Studies that include victims or witnesses of wrongdoing, however, will not be eligible. Thus, only the data relevant to mock suspects will be considered if a study population comprises more than mock suspects. However, the type of mock‐suspect will not be limited by race, age, ethnicity, gender, or any other demographic characteristics.

#### 3.1.3 Types of interventions

For the purposes of this partial update, an interview or interrogation method is (1) an intentional use of one or more (2) established interrogation tactics used to (3) induce a confession. An intentional use means that the intervention is part of the experimental manipulations (see Types of Studies) and that at least part of the interrogation tactic was scripted. In other words, studies where mock experimenters were allowed complete freedom in how they attempted to get a confession will not be eligible. By established interrogation tactic, we mean those tactics that have been associated with either an accusatorial or information‐gathering approach. For example, false evidence ploys are associated with accusatorial methods and are included in accusatorial interrogation manuals (Inbau et al., 2013). When not identified by name, an accusatorial technique can be identified by their goal: to obtain a confession, typically modeled on the Reid technique (see Inbau et al., 2001). Accusatorial techniques also include the use of minimization and maximization tactics, which themselves encompass a wide range of more specific tactics (see Kelly et al., 2015 for an overview). Conversely, information‐gathering techniques can be identified by their goal to seek information, an example being the PEACE model (see Milne & Bull, 1999). Information‐gathering techniques often use cognitive interview and rapport‐building tactics. Any tactic identified by Gabbert et al. (2021) would be eligible. Direct questioning or control techniques can involve elements of the other two techniques, but the main advance of control techniques are short, declarative sentences/questions that are goal‐oriented. Finally, to determine that a tactics' purpose is to induce a confession, the tactic must be introduced before the request for a confession.

#### 3.1.4 Types of outcome measures

##### Primary outcomes

Eligible studies will include either true or false (or both) confession rates as the dependent variable. Depending on the study design (e.g., some studies include exclusively innocent participants), the study must report either the number of true confessions (i.e., confessions provided by guilty participants), false confessions (i.e., confessions provided by innocent participants), or both. The ground truth of the confession (i.e., true vs. false confession) is the priority for the coding stage. If a study contains both primary confession and secondary confession information, only the primary confession data will be considered. Primary confessions are those provided by the supposed wrongdoer and are the outcome of interest. Secondary confessions are typically defined as either an individual admitting they witnessed an act of wrongdoing, which could make them complicit because of their lack of action (e.g., Swanner et al., 2010), or an individual conveying that the supposed wrongdoer confessed to them (e.g., Wetmore et al., 2014). In either case, the individual is not the supposed wrongdoer and will not be considered in our analyses.

##### Secondary outcomes

There are no secondary outcomes for this review.

#### 3.1.5 Duration of follow‐up

This is not relevant for the review. The dependent variable is measured during the single laboratory session with the subject.

#### 3.1.6 Types of settings

There is no proposed geographic limitation to study location. However, for pragmatic reasons, only studies published in English will be considered.

### 3.2 Search methods for identification of studies

#### 3.2.1 Electronic searches

The listed databases and keywords are heavily influenced by the meta‐analysis being replicated (Meissner et al., 2012). The keyword combinations and filters, however, were generated by the first author of the current effort. We will search across titles, abstracts, author‐supplied keywords, and indexing terms when possible. All searches will be limited to those results available in English. We will not restrict our search or eligibility criteria by the date of publication as all relevant studies, even those captured in the original effort, will be included in our analyses. The following databases, organized by publisher platforms, are proposed for the search process:

ProQuest

1. Australia & New Zealand Database
2. Criminal Justice Database
3. ERIC
4. ProQuest Dissertations & Theses Global
5. Psychology Database
6. Social Science Database
7. Sociological Abstracts
8. Sociology Database
9. UK & Ireland Database

EBSCO

1. APA PsycExtra
2. APA PsycInfo
3. Criminal Justice Abstracts
4. National Criminal Justice Reference Services Abstracts
5. Psychology and Behavioral Sciences Collection

Web of Science

1. Conference Proceedings Index: Social Sciences & Humanities
2. Social Science Citation Index

Other

1. CINCH: Australian Criminology Database (Informit)
2. Google
3. Google Scholar

The following keywords will be used in the systematic search (* denotes that the word could have various endings; for example, interrogat* could be interrogation or interrogator):

1. Interrogat*
2. Information (gathering)
3. Inquisitorial
4. Interview*
5. Suspect*
6. Confess*
7. ‘Cognitive interview*’
8. ‘Conversation management’
9. ‘Ethical interview*’
10. Disclos*
11. ‘Strategic evidence’
12. Accusat*
13. ‘Deception detection’
14. PEACE
15. PACE
16. Adversar*
17. Miranda
18. Coerc*
19. Entrap*
20. ‘Due process of law’
21. Reid
22. Minimi?*
23. Maximiz*
24. Random*
25. Control
26. Comparison
27. Experiment*
28. RCT
29. Manipulat*
30. Lab*
31. Factorial
32. Guilt*
33. Innocen*
34. Responsib*
35. Commit*
36. Effect

The keywords will be condensed to the following search logic for databases that allow for advanced Boolean logic: (interrogat* OR information OR inquisitorial OR interview* OR accusat* OR “deception detection” OR PEACE OR PACE OR adversar* OR REID OR minimi?* OR maximiz* OR “cognitive interview*” OR “conversation management” OR “ethical interview*” OR “strategic evidence” OR miranda OR coerc* OR entrap* OR responsib* OR commit*) AND (random* OR control OR comparison OR experiment* OR RCT OR manipulat* OR lab* OR factorial OR effect) AND (confess* OR disclos*) AND (suspect* OR guilt* OR innocen*). See Supporting Information: Appendix 1 for the full search conducted in ProQuest.

#### 3.2.2 Searching other resources

Once the digital search has been completed, all studies deemed eligible for inclusion will be searched for relevant literature. Each eligible study will also be used to conduct forward citation searching in Google Scholar. Furthermore, we have several resources to complement the search, including:

1. Meissner, C. A., Redlich, A. D., Bhatt, S., & Brandon, S. (2012). Interview and interrogation methods and their effects on true and false confessions. *Campbell Systematic Reviews*. doi:10.4073/csr.2012.13 [Lists approximately 80 references]
2. Madon, S., More, C., & Ditchfield, R. (2019). Interrogations and confessions. In N. Brewer & B. Douglass (Eds.) *Psychological science and the law*. New York: Guilford Press. [Lists approximately 75 references]
3. Kassin, S. M., Drizin, S., Grisso, T., Gudjonsson, G., Leo, R. A., & Redlich, A. D. (2010). APLS Approved White Paper, Police‐induced confessions: Risk factors and recommendations. *Law and Human Behavior*. doi:10.1007/s10979-009-9188-6. [Lists approximately 300 references]
4. Stewart, J. M., Woody, W. D., Pulos, S. (2016). The prevalence of false confessions in experimental laboratory simulations: A meta‐analysis. *Behavioral Sciences & the Law, 36*, 12–31. [Lists approximately 100 references]
5. High‐Value Detainee Interrogation Group. (2016). *Interrogation: A review of the Science*. [Lists approximately 780 references]

Finally, the reviewers have many well‐established contacts with researchers studying interviewing and interrogation here in the United States and abroad. In Table 1, we have started a list of possible researchers to contact. We will reach out to known and unknown contacts for unpublished or “in press” studies to possibly include.

**Table 1:** Initial list of potential individuals to contact.

Name	Affiliation
Iris Blandón‐Gitlin	California State University, Fullerton, FL, USA
Randy Borum	University of South Florida, FL, USA
Joseph Buckley	John Reid and Associates, IL, USA
Stephanie Cardenas	Williams College, MA, USA
Julie Cherryman	University of Portsmouth, United Kingdom
Mark Costanzo	Claremont Graduate School, CA, USA
Brian Cutler	Ontario Tech University, Canada
David Dixon	University of New South Wales, Australia
Steven Drizin	Northwestern School of Law, IL, USA
Jacqueline Evans	Florida International University, FL, USA
Ronald Fisher	Florida International University, FL, USA
Par Anders Granhag	Goteborg University, Sweden
Max Guyll	Iowa State University, IA, USA
Maria Hartwig	John Jay College of Criminal Justice, NY, USA
Lorraine Hope	University of Portsmouth, United Kingdom
Saul Kassin	John Jay College of Criminal Justice, NY, USA
Mark Kebbell	Griffith University
Christopher Kelly	St. Joseph's University, PA, USA
Steven Kleinman	MacDill Air Force Base, FL, USA
Gunther Kohnken	University of Kiel, Germany
Jeff Kukucka	Towson University, MD, USA
Michael Lamb	University of Cambridge, United Kingdom
Amy Leach	Ontario Tech University, Canada
Richard Leo	University of San Francisco School of Law, CA, USA
Stephanie Madon	Iowa State University, IA, USA
Samantha Mann	University of Portsmouth, United Kingdom
Jaume Masip	University of Salamanca, Spain
Rebecca Milne	University of Portsmouth, United Kingdom
Amelia Mindthoff	Iowa State University, IA, USA
Fadia Narchet	University of New Haven, CT, USA
Christopher Normile	Allegheny College, PA, USA
Jennifer Perillo	Indiana University of Pennsylvania, PA, USA
Melissa Russano	Roger Williams University, RI, USA
Kyle Scherr	Central Michigan University, MI, USA
Laura Smalarz	Arizona State University, AZ, USA
Brent Snook	Memorial University of Newfoundland, Canada
Leif Stromwall	University of Gothenburg, Sweden
Paul Taylor	Lancaster University, United Kingdom
Aldert Vrij	University of Portsmouth, United Kingdom

### 3.3 Data collection and analysis

#### 3.3.1 Description of methods used in primary research

The typical study will randomly assign mock suspects (volunteers, typically college/university students) to one of two or more experimental conditions representing different interrogation methods. The two most common experimental paradigms are referred to as the cheating paradigm (e.g., Russano et al., 2005) and the alt‐key paradigm (e.g., Kassin & Kiechel, 1996). In the cheating paradigm, interrogation methods are typically crossed with guilt status. Guilt status is manipulated by a confederate who either does or does not induce participants to help them solve an independent logic problem. In the alt‐key paradigm, all participants are typically innocent as the computer is programmed to crash regardless of participant action. Regardless of guilt, all participants will be accused of some wrongdoing. These mock suspects can be entirely aware of the nature of the study (e.g., some studies challenge participants to “get away” with an act of wrongdoing), but are typically deceived to various degrees (e.g., some studies lead participants to believe they are facing academic consequences for the supposed wrongdoing). At the point of accusation, studies typically manipulate the interrogation method through experimenter scripts before asking for a signed confession from participants (see Stewart et al., 2016 for an overview of typical experimental confession studies).

Based on our knowledge of the field, we do not anticipate eligible studies will include non‐standard designs. If, however, a non‐standard design is discovered, a member of our research team is an expert in meta‐analysis and works closely with the Campbell Collaboration and they will be responsible for calculating effect sizes from non‐standard designs.

#### 3.3.2 Selection of studies

Two independent coders will screen the titles and abstracts, using Abstrackr (Abstrackr.cebm.brown.edu), of all studies identified in the digital search for potential eligibility. Each coder will screen 100% of the titles and abstracts and inter‐rater reliability will be evaluated by percent agreement. The independent coders will be trained by the first author by going over the pre‐registered protocol, practicing screening a subset together, and then going over a pilot round of abstracts screened independently. Furthermore, the coders and first author will meet on a weekly basis to discuss any confusions or disagreements as they arise. All studies deemed potentially eligible (i.e., both raters answered ‘yes’ or ‘maybe’ when asked if the study meets all eligibility criteria) will then be accessed in their full form against the eligibility criteria. This second round of screening will also be conducted by two independent coders who will determine final eligibility. A single individual will scan the reference lists of eligible studies and of the secondary search sources, such as prior reviews.

#### 3.3.3 Data extraction and management

All studies deemed eligible for inclusion will be coded for key variables (e.g., effect size information) and study characteristics (e.g., publication type) by two independent coders. Discrepancies will be resolved through discussion, and when consensus cannot be reached, one of the lead reviewers will make the final decision.

Coders will be trained by the lead reviewers in steps: (1) coders will verbally walk through the code sheet for discussion and clarification, (2) the lead reviewers will demonstrate how to code an article in its entirety, and (3) the coders will practice on a small subset of articles for review and feedback from the lead reviewers. This iterative process will continue throughout the coding process with regular meetings to discuss coding issues.

Coders are likely to be volunteers from Dr. Redlich's research team, some of whom have prior experience with meta‐analyses. Generally, coding will include four hierarchical data levels: a study level, an experimental condition level, an outcome level, and an effect size level. Using LibreOffice, we will create a database that allows for the one‐to‐many hierarchical nature of our coding protocol (e.g., one study could include several experimental conditions, measure more than one outcome, and have several effect sizes).

- Study level variables will include static information (e.g., publication type, publication year, geographic location). As such, there will be one record per study at this level of coding.
- Experimental condition level coding will be conducted for each relevant group of the research design. Thus, there will be one record for each eligible experimental condition within a study. For example, if a study included a factor with three levels of interrogation techniques (i.e., accusatorial vs. information‐gathering vs. direct questioning), three condition coding sheets will be completed to capture each group. Information specific to each condition will be coded at this level, such as sample size and interrogation method.
- The outcome level will code information specific to each eligible outcome measure. Thus, there will be one record per outcome. In addition to indicating the outcome construct, coded items will capture whether the variable includes all participants, innocent participants, guilty participants, or some other grouping.
- The effect size level will code all necessary statistical information to calculate a logged odds ratio (L_OR_) and its variance for each outcome. As such, these will be one record per coded effect size. Coders will be instructed to identify the most detailed numerical data available when coding for effect size information (see Supporting Information: Appendix 2 for the full coding sheet). When eligible studies do not report all necessary data, we will make a good faith effort to contact authors to obtain the necessary information.

A subset of these studies will be used to test the initial coding protocol. That is, the coding protocol will be tested for usability/clarity and utility in capturing relevant information from each study. This initial testing phase of the coding protocol will also provide an additional training opportunity for the coders. We anticipate that the initial coding will result in refinements to the coding protocol to ensure consistent coding across coders and alignment between coding options and study characteristics.

#### 3.3.4 Assessment of risk of bias in included studies

We will assess the risk of bias of the included studies through a combination of unique coding items developed by us specifically for this research literature and items that were adapted from the Cochrane risk‐of‐bias tool for randomized trials (Higgins et al., 2019). The language of the latter were modified to better fit the characteristics of studies eligible for this review. We excluded items that were not relevant to this literature. The specific items are in the coding protocol (see Supporting Information: Appendix 2).

More specifically, the risk‐of‐bias items address the following methodological issues: random assignment to both the interrogation technique and guilt conditions, treatment of violations to the randomization process (e.g., participants assigned to the guilty condition who refused to cheat), level of deception employed in the study, treatment of participants suspicious of the true purpose of the study, and whether mock interrogators were blind to the guilt status of mock suspects. To address the confession outcome, coders will document any missingness of confession outcomes, including selective reporting of confession outcomes. See Supporting Information: Appendix 2 for the coding protocol.

We will provide a table of these items for each coded study in the final report. Furthermore, we will investigate the potential for bias by examining the relationship between each bias item and effect sizes in a moderator analysis. Potential sources of bias and the associated moderator analyses will inform our interpretation of the findings.

#### 3.3.5 Measures of experimental effect

We will use the odds ratio as the effect size index. The outcomes are dichotomous and we are interested in comparing pairs of experimental conditions. Thus, the data can be represented as a 2 × 2 contingency table.

#### 3.3.6 Unit of analysis issues

The unit of analysis will be the individual study participant. We do not anticipate any complex issues around the unit‐of‐analysis or unit‐of‐assignment (also the participant) among the eligible designs.

#### 3.3.7 Criteria for determination of independent findings

There are only two eligible outcomes for this review: true confession and false confession. However, because these studies may have any number of experimental conditions, numerous effect sizes may be possible for each outcome. For example, if a study has three conditions (accusatory, information‐gathering, and direct questioning), there are three possible pairings of these conditions and as such three possible effect sizes for each outcome. We will code all possible effect‐size combinations but maintain independence at the analysis stage in two ways. The first is simply to perform separate meta‐analyses for each contrast of interest, such as an analysis comparing accusatory to information‐gathering methods. If a study has two accusatory conditions, these will be collapsed for such an analysis. The second is to perform a network meta‐analysis that takes advantage of the network of comparisons provided by these studies.

Given the nature of the research designs in this area and based on the previous work done on this topic (i.e., Meissner et al., 2012), we do not anticipate that any study will employ repeated measurement of an outcome of interest. Thus, we do not anticipate outcomes from multiple timepoints to be problematic.

#### 3.3.8 Dealing with missing data

We will contact authors to request missing effect‐size data. Any study that meets all eligibility criteria but for which we are unable to compute an effect size and are unable to get the needed data from the authors will be identified and discussed in the manuscript. Missing descriptive information regarding a study's methods will simply be noted and reported.

#### 3.3.9 Assessment of heterogeneity

We will assess heterogeneity using the *Q*‐test and the *I* ^2^ statistic.

#### 3.3.10 Assessment of reporting biases

Given the nature of these studies, selective outcome reporting is unlikely. It is common practice for some researchers to only measure one of the two outcomes of interest. However, we note as part of our risk‐of‐bias tool if there is any indication in a coded manuscript that one of the two outcomes of interests was measured but not reported. The greater risk in this literature is publication bias, though we will attempt to mitigate this risk by purposefully seeking unpublished work as well as published manuscripts. When there are at least 10 effect sizes for a given analysis, publication bias will be assessed. We will do so in three ways: (1) a visual inspection of the funnel plot; (2) a trim‐and‐fill analysis, including reporting the adjusted effect size estimate; and (3) an Egger's regression test.

#### 3.3.11 Data synthesis

Data synthesis will be conducted via random‐effects meta‐analysis based on the logged odds ratio. The models will be estimated using the restricted maximum likelihood (REML) estimator of *τ* ^2^. The basic meta‐analyses will be run using the *metafor* package by Viechtbauer (Viechtbauer, 2010). As stated above, separate models will be estimated for each conceptually relevant pairing of interview style.

We will also conduct a network meta‐analysis. A network meta‐analysis extends a traditional meta‐analysis by examining all available comparisons (both direct and indirect) in a network. A network meta‐analysis is particularly suited to our goal as it will allow us to directly compare the relative effectiveness of interrogation techniques. Within this network, we will differentiate variations on the interview method, such as maximization and minimization accusatory approaches. This separation is important to investigate as some studies have found that minimization tactics increase confessions (Guyll et al., 2019; Normile & Scherr, 2018), while others find no differences in direct‐questioning and minimization tactics (Woestehoff, 2016). Following suggestions from the Campbell methods brief on network meta‐analysis, we will present a network diagram, information on inconsistency factors, a league table, and ranking of each interrogation approach through rankograms and cumulative ranking plots (see Wilson et al., 2016). The network meta‐analyses will be conducted in R using the *netmeta* package (Rücker et al., 2021).

#### 3.3.12 Subgroup analysis and investigation of heterogeneity

We will perform categorical moderator analyses on the cheating paradigm versus the alt‐key paradigm. This will be performed using the *metafor* package by Viechtbauer (Viechtbauer, 2010) and the analog‐to‐the‐ANOVA analytic framework. In metafor, this is accomplished via the rma.uni() function combined with the predict() function. This approach assumes a common *τ* ^2^ across subgroups, an assumption that seems reasonable for these studies. Likewise, we will conduct moderator analyses using each risk of bias item to determine how potential bias will influence the interpretation of our results.

#### 3.3.13 Sensitivity analysis

We will be analyzing effect sizes in two ways. First, using traditional meta‐analytic methods to examine each experimental condition pairing of interest. Second, using network meta‐analysis. These two approaches are complementary and provide a form of sensitivity analysis of each method.

#### 3.3.14 Treatment of qualitative research

Qualitative research will not be considered as part of this review.

#### 3.3.15 Summary of findings and assessment of the certainty of the evidence

We will provide a *Summary of Findings* table with the results of the meta‐analyses. We will not, however, use GRADE or a GRADE‐like system as we do not believe that it is appropriate for this review. The focus here is not an assessment of whether a treatment is effective or ineffective. Rather, we are trying to establish the relative performance of different interview methods in eliciting reliable versus unreliable confessions. The confidence intervals around the mean effect sizes are the first line of information on the certainty of the evidence. The second line of information on the certainty of the evidence is methodological weaknesses identified via our risk‐of‐bias assessment. The overall results will be interpreted within the context of any weaknesses identified, particularly if they are prevalent across studies.

## CONTRIBUTIONS OF AUTHORS

Mary Catlin is an advanced graduate student being advised by Professor Redlich. She has conducted a pilot test of the updated meta‐analysis outlined above as part of a class assignment for a meta‐analysis course taught by Professor Wilson, who is an expert in meta‐analysis. Professor Wilson has written one of the most widely used texts on meta‐analysis (Lipsey & Wilson, 2001), serves as the methods editor for the Crime and Justice Group for the Campbell Collaboration, is an associate editor for Research Synthesis Methods, and was awarded the Frederick Mosteller Award for Distinctive Contributions to Systematic Reviewing. Furthermore, Professor Wilson has produced several systematic reviews within the field of criminology (e.g., Wilson et al., 2018, 2019) and has written on the utility of network meta‐analyses with recommendations for Campbell reviews specifically (Wilson et al., 2016).

Both Professors Redlich and Meissner are content experts and conducted the original review. Professor Redlich has reviewed the literature on US police and military interrogations, most notably as part of the editing team for two volumes outlining investigative interviewing and interrogation internationally (Walsh et al., 2016a, 2016b, 2016). Furthermore, Professor Redlich was involved in the American Psychology‐Law Society's scientific review committee's “white paper” on police interrogations and false confessions (see Kassin et al., 2010). Professor Meissner has evaluated the deception detection and interviewing/interrogation literatures, including conducting several meta‐analyses in this area (Meissner & Kassin, 2002; Meissner et al., 2017; Snook et al., 2021). He has also co‐organized a conference sponsored by the American Psychological Association on investigative interviewing. This conference developed into a co‐edited volume entitled, Interrogations and confessions: Current research, practice, and policy recommendations, which was published by the American Psychological Association (Lassiter and Meissner, 2010). Furthermore, Professor Meissner was awarded the Excellence in Research Award from the International Investigative Interviewing Research Group, has been awarded a 3 year research grant from the US Department of Justice to investigate the state of the interrogation literature and the dissemination of scientific knowledge in that area.

Talley Bettens is a graduate student being supervised by Professor Redlich. She has assisted Professor Redlich with another meta‐analysis, currently under review for publication, that required many of the same tasks/skills that will be asked of her for the current endeavor.

Finally, both Sujeeta Bhatt and Susan Brandon contributed to the original review. Furthermore, Drs. Bhatt and Brandon served as program managers for research at the High‐Value Detainee Interrogation Group (Federal Bureau of Investigation). Under their leadership, a decade of research was conducted on best practices in interviewing and interrogation. In this context, Drs. Bhatt and Brandon have contributed significantly to the development of the research literature on science‐based approaches to investigative interviewing (Brandon & Meissner, in press; Brandon et al., 2019).

- Content: Mary Catlin and Allison Redlich
- Systematic review methods: Mary Catlin and David B. Wilson
- Statistical analysis: Mary Catlin and David B. Wilson
- Information retrieval: Mary Catlin and Christian A. Meissner
- Screening and coding: Mary Catlin and Talley Bettens
- Drafting of reports: Mary Catlin, Allison Redlich, David B. Wilson, and Christian A. Meissner
- Editing of reports: all authors

## DECLARATIONS OF INTEREST

None of the reviewers have financial conflicts of interest. Drs. Redlich, Meissner, Bhatt, and Brandon were authors on the original review which this update is based on. Furthermore, Professor Wilson was the Campbell editor who oversaw the original review.

## PRELIMINARY TIMEFRAME

**Table:** 

Searches for published and unpublished studies:	September 1, 2021–October 31, 2022
Pilot testing and coding:	January 1, 2022–May 1, 2022
Analyses:	September 1–January 1, 2023
Preparation of final report:	November 15, 2022–March 15, 2023
Dissemination and publication:	March 16–May 30, 2023

## PLANS FOR UPDATING THIS REVIEW

The review will be updated every 5–10 years. These efforts will primarily be led by reviewers Meissner and Redlich, or their students.

## SOURCES OF SUPPORT

**Internal sources**

- No sources of support provided

**External sources**

- High‐Value Detainee Interrogation Group, USA

HIG is a three‐agency entity (FBI, CIA, DOD) dedicated to evidence‐based interrogation practices. They fund researchers to test and identify effective interrogation approaches. To that end, HIG—through Iowa State University—is funding the current project in exchange for a report (submitted September 2022) outlining which interrogation approaches are most effective at maximizing true confessions and minimizing false confessions.

## Supporting information

Supporting information.
