# Supplementary material for: PROTOCOL: Interview and interrogation methods and their effects on true and false confessions: An update and extension
Source: Campbell Syst Rev. 2023 Mar 2;19(1):e1314. doi: 10.1002/cl2.1314 (PMC9979963; doi:10.1002/cl2.1314)
Supplement: Supplementary file 1 — Supporting information. [file CL2-19-e1314-s001.docx]

Appendices

1 ProQuest Search Strategy

ProQuest Search Strategy: October 20, 2022

| **#** | **Searches** | **Databases** | **Results** |
| --- | --- | --- | --- |
| 1 | AB,TI,IF,CC(interrogat* OR information OR inquisitorial OR interview* OR accusat* OR ‘deception detection’ OR PEACE OR PACE OR adversar* OR REID OR minimi?* OR maximiz* OR ‘cognitive interview*‘ OR ‘conversation management’ OR ‘ethical interview*‘ OR ‘strategic evidence’ OR miranda OR coerc* OR entrap* OR responsib* OR commit*) | Australia & New Zealand Database, Criminal Justice Database, ERIC, ProQuest Dissertations & Theses Global, Psychology Database, Social Science Database, Sociological Abstracts, Sociology Database, UK & Ireland Database | 2,635,350 |
| 2 | AB,TI,IF,CC(random* OR control OR comparison OR experiment* OR RCT OR manipulat* OR lab* OR factorial OR effect) | Australia & New Zealand Database, Criminal Justice Database, ERIC, ProQuest Dissertations & Theses Global, Psychology Database, Social Science Database, Sociological Abstracts, Sociology Database, UK & Ireland Database | 4,348,167 |
| 3 | AB,TI,IF,CC(confess* OR disclos*) | Australia & New Zealand Database, Criminal Justice Database, ERIC, ProQuest Dissertations & Theses Global, Psychology Database, Social Science Database, Sociological Abstracts, Sociology Database, UK & Ireland Database | 77,042 |
| 4 | AB,TI,IF,CC(suspect* OR guilt* OR innocen*) | Australia & New Zealand Database, Criminal Justice Database, ERIC, ProQuest Dissertations & Theses Global, Psychology Database, Social Science Database, Sociological Abstracts, Sociology Database, UK & Ireland Database | 126,325 |
| 5 | 1 and 2 and 3 and 4 | Australia & New Zealand Database, Criminal Justice Database, ERIC, ProQuest Dissertations & Theses Global, Psychology Database, Social Science Database, Sociological Abstracts, Sociology Database, UK & Ireland Database | 532 |

## 2 Initial Coding Protocol

**Study Level Coding Form**

Please complete each field to the best of your ability. If you have any comments, questions, or concerns, please include those in the notes section.

Please fill out 1 Study-Level form per study. For our purposes, a study is identified as an experiment with an independent sample. Some publications may include more than one study (e.g., multiple independent experiments) and thus will require a form for each.

1. [s_id] Study ID

*--8 digits. Should match the file name in most cases. If it is the case that the same publication has several studies, see the definition of a study above, add sequential lettering at the end (e.g. 00001234a, 00001234b)*

2. [s_coder] Coder’s initials

*--Study level coder’s initials (e.g., MC).*

**Study header information**

3. [s_ref] Reference

*-- List of authors, last names only (e.g., Redlich & WIlson). If more than 2 authors, use et al. format (e.g., Bettens et al.)*

4. [s_year] Year of publication

*-- Year of publication (YYYY).*

5. [s_pubtype] Publication type

1. Journal article

2. Book

3. Book chapter

4. Thesis/dissertation

5. Technical report

6. Conference paper

7. Other

*--If more than one is applicable, choose the lowest numbered option (e.g., a dissertation later published as a book would be coded as a book)*

6. [s_loc] Geographic location of the study

1. United States

2. Canada

3. United Kingdom

4. Australia

5. Other

*-- Location where the data was collected.*

**Study Methodology**

7. [s_para_label] What did the authors label the paradigm?

8. [s_para] Paradigm

1. Cheating paradigm

2. Alt-key paradigm

3. Mock crime

4. Other

*--What paradigm was used in this study? Use other for “novel” paradigms with no further clarification.*

9. [s_conf] How was confession measured?

1. Yes-no

2. Resistance

3. Compliant

4. Internalized

5. Confabulated

6. Other

*--Indicate each way the authors indicated the confession outcome was conceptualized*

10. [s_attempts] How many times were mock-suspects asked to confess?

1. Once

2. Twice

3. Three times

4. More than 3 times

5. Unclear

11. [s_suspect] Suspect identity

1. College students

2. Juveniles

3. Community members

4. Combination

5. Other

6. Unclear

*--Who were the mock-suspects? What population were participants recruited from?*

12. [s_interrogator] Interrogator identity

1. Students

2. Professor

3. Practitioners

4. Combination

5. Unclear

*--Who ran the sessions? Who administered the treatment/interrogation?*

13. [s_notes] Notes about general study characteristics

**Risk of Bias Coding Form**

Please complete each field to the best of your ability. If you have any comments, questions, or concerns, please include those in the notes section.

Please fill out 1 Study-Level form per study. For our purposes, a study is identified as an experiment with an independent sample. Some publications may include more than one study (e.g., multiple independent experiments) and thus will require a form for each.

1. [s_id_rob] Study ID

*--8 digits. Should match the file name in most cases. If it is the case that the same publication has several studies, see the definition of a study above, add sequential lettering at the end (e.g. 00001234a, 00001234b)*

2. [s2_coder] Coder’s initials

*--Study level coder’s initials (e.g., MC).*

**Questions concerning randomization**

3. [s2_rob1] Were participants randomly assigned to interrogation technique conditions?

1. Yes

2. No

3. Unclear

4. [s2_rob2] Were participants randomly assigned to guilt conditions?

1. Yes

2. No

3. Unclear

4. Not applicable

-->5. [s2_rob2] Were mock interrogators blind to the guilt status of the mock suspects?

1. Yes

2. No

3. Unclear

**Questions concerning attrition**

6. [s2_sample] Total starting sample size

*-- Number of participants recruited before any attrition (999 if unclear or unknown)*

7. [s2_excluded] Number of excluded participants

*-- Total number of participants excluded from analysis, regardless of reason (999 if unclear or unknown)*

8. [s2_usable] Total analytic sample size

*-- Total number of usable participants (999 if unclear or unknown)*

9. [s2_attrition]

| Explanations for differences in original sample size and sample analyzed | | | | |
| --- | --- | --- | --- | --- |
|  | Addressed | n | Included in analyses | Notes |
| Violation of randomization |  |  |  |  |
| Suspicious |  |  |  |  |
| Mock-interrogator differences |  |  |  |  |
| Other |  |  |  |  |

*DIRECTIONS:*

*“Addressed” should be a ‘yes’ or no’ response dependent on whether the authors considered (i.e., reported in the manuscript) each respective reason for participant attrition or exclusion.*

*“n” should reflect the number of participants or data points associated with each exclusion rationale as reported by the authors (999 if unclear or unknown).*

*“Included in analyses” should be a ‘yes’ or ‘no’ response to indicate whether these data were included in the final analyses.*

*“Notes” should indicate anything of note for each rationale. For example, you should indicate if violations of randomization were analyzed as assigned. For “Other”, you could indicate what the reason for excluding these participants was.*

**Other considerations**

10. [s2_rob4] What level of deception (i.e., awareness of accusation/true purpose of the study) was employed in the study?

1. Participants were completely unaware of the true purpose of the study

2. Participants were partially aware of the true purpose of the study

3. Participants were completely aware of the true purpose of the study

4. Unclear

11. [s2_rob8] Were mock-interrogators’ interrogation scripted?

1. Yes

2. No

3. Unclear

12. [s2_rob9] Did researchers report outcomes for every way in which they measured confession?

1. Yes

2. No

3. Unclear

*-- For example, if a study considered compliant, internalized, and confabulation were all three types of confession outcomes reported?*

13. [s2_notes] Notes about risk of bias

**Condition Level Coding Form**

Please complete each field to the best of your ability. If you have any comments, questions, or concerns, please include those in the notes section.

Please fill out 1 form per unique condition. For example, if a study has 2 experimental groups and 1 comparison group, then 3 unique condition forms are necessary.

You should code each condition in the order that they are addressed in the manuscript. For example, if an author states that they had 3 levels of interrogation approach (accusatorial vs. information vs. control), then you should code the accusatorial condition, then information condition, and finally the control condition.

1. [s_id_c] Study ID

*--8 digits. Should match the file name in most cases. If it is the case that the same publication has several studies, see the definition of a study above, add sequential lettering at the end (e.g. 00001234a, 00001234b)*

2. [c_id] Condition ID

*--3 digits. Should be a unique ID (e.g., 001, 002, 00k, within each study)*

3. [c_coder] Coder’s initials

*--Study level coder’s initials (e.g., MC).*

**Description of condition**

4. [c_label] What did the authors call this technique?

5. [c_approach] Interrogation approach

1. Reid/Accusatorial interrogation technique

2. PEACE/Information-gathering technique

3. Control or direct questioning

*--What approach was this group exposed to?*

**-->**6. [c_reid] Characteristics of accusatorial manipulation

1. Maximization techniques

2. Minimization techniques

3. Both minimization and maximization techniques

4. Evidence ploy

5. Other

6. Unclear

7. Missing

*--What tactic under accusatory methods was the experiment modelled after?*

**Demographic characteristics of condition**

7. [c_race]

Racial/Ethnic breakdown

% Source

White

Black

Latinx

Asian

Other

*DIRECTIONS:*

*“%“ = percent of this group that is represented by each of the following race/ethnic groups (999 if unclear or unknown).*

*“Source” = the group that the percentage is based off of. Specifically, is the % derived from the condition, the full sample before attrition, the analytic sample, or unclear?*

8. [c_sex_n] Sex distribution

*--% male (999 if unclear or unknown)*

9. [c_sex_source] Sex (source)

1. Condition only

2. Full sample before attrition

3. Full sample after attrition/analytic sample

4. Unclear

*--What group is this percentage based on?*

10. [c_age] Mean or median age

*--999 if unclear or unknown*

11. [c_age_source] Age (source)

1. Condition only

2. Full sample before attrition

3. Full sample after attrition/analytic sample

4. Unclear

*--What group is this percentage based on?*

13. [c_notes] Notes about condition

**Outcome (Dependent Variable) Coding Form**

Please complete each field to the best of your ability. If you have any comments, questions, or concerns, please include those in the notes section.

Please fill out 1 form per outcome (i.e., dependent variable). For example, if a study measures confessions for both innocent and guilty participants, then 2 outcome forms are necessary.

To order your outcome IDs, always start with false confessions then true confessions.

1. [s_id_o] Study ID

*--8 digits. Should match the file name in most cases. If it is the case that the same publication has several studies, see the definition of a study above, add sequential lettering at the end (e.g. 00001234a, 00001234b)*

2. [o_id] Outcome ID

*--3 digits. Should be a unique ID for each outcome (e.g., 001, 002, 00k) within each study*

3. [o_coder] Coder’s initials

*--Study level coder’s initials (e.g., MC).*

4. [o_label] What did the researchers label this outcome?

*--For example, did the researchers refer to this outcome as innocent confessions, false confessions, super innocents, etc.?*

5. [o_dv] Dependent variable type

1. False confessions

2. True confessions

3. Other

4. Unclear

*--If the outcome is measured for some other group, indicate what that group is in the notes section.*

6. [o_notes] Notes about DV

**Effect Size Coding Form**

Please complete each field to the best of your ability. If you have any comments, questions, or concerns, please include those in the notes section.

Please fill out 1 form per effect size, keep as many decimal places as reported by the study authors. Furthermore, data for calculating ES only needs to be submitted once and should include the most detailed numerical data available in the study. In other words, if you have the 2 X 2 frequency table information and a phi correlation, you should only record the raw frequencies identified in the 2 X 2 frequency table. Always default to raw frequencies if provided with more than one way to calculate the ES. Order of preference is as follows (most preferred ES to least): raw frequencies, proportions without inference, proportions with inference, chi square or phi coefficient without inference, chi square or phi coefficient with inference, and Cohen’s d.

1. [s_id_es] Study ID

*--8 digits. Should match the file name in most cases. If it is the case that the same publication has several studies, see the definition of a study above, add sequential lettering at the end (e.g. 00001234a, 00001234b)*

2. [c_id1_es] Condition ID 1

*--3 digits. Should be the ID representing the group to be treated as the experimental condition in this contrast. This connects this ES data to the appropriate conditional level coding form*

2. [c_id2_es] Condition ID 2

*--3 digits. Should be the ID representing the group to be treated as the control or contrasting condition. This connects this ES data to the appropriate conditional level coding form*

2. [o_id_es] Outcome ID

*--3 digits. Should be the ID for the outcome associated with this ES. This connects this ES data to the appropriate outcome level coding form*

3. [es_coder] Coder’s initials

*--Study level coder’s initials (e.g., MC).*

4. [es_type] ES calculation type

1. 2X2 frequency

2. Binary proportions

3. Phi coefficient

4. Chi square

5. Cohen’s d

5. [es_freq]

2X2 Frequency

Confession Denial

Experimental Group

Control Group

6. [es_prop]

Proportions

Sample size (per condition) Proportion of Confession (per condition)

Experimental

Control

7. [es_phi]

| Phi coefficient (r) | Proportion of full sample that confessed | Condition 1 sample size | Condition 2 sample size |
| --- | --- | --- | --- |
|  |  |  |  |

8. [es_chi]

| Chi-square statistic | Proportion of full sample who confessed | Condition 1 sample size | Condition 2 sample size |
| --- | --- | --- | --- |
|  |  |  |  |

9. [es_d]

| Cohen’s d | Variance of Cohen’s d | Condition 1 sample size | Condition 2 sample size | Code for calculating ES |
| --- | --- | --- | --- | --- |
|  |  |  |  |  |

10. [es_n_infer] How did you infer the sample sizes for these conditions?

11. [es_pg] Page where effect size data was found

12. [es_notes] Notes about this effect size

*--In addition to any other commentary, use this section to indicate if it was not possible to complete ES data or if certain figures needed to be imputed and how.*
